# Supplementary material for: Overexpression of Auxin/Indole-3-Acetic Acid Gene TrIAA27 Enhances Biomass, Drought, and Salt Tolerance in Arabidopsis thaliana
Source: Plants (Basel). 2024 Sep 25;13(19):2684. doi: 10.3390/plants13192684 (PMC11478388; doi:10.3390/plants13192684)
Supplement: Supplementary file 1 [file plants-13-02684-s001.zip › plants-2950376-supplementary.pdf]

Supplementary Materials

Table S1. Primers for PCR and qRT-PCR

Name	Primer's sequence
TrIAA27-F	5'-ATGTCTGTGCCAGTGGAAC-3'
TrIAA27-R	5'-CTAGTTGCGACTTTTGCACT-3'
TrIAA27-RACE3'	5'-CCAAATGCATAAGCCATGCCATGCC-3'
TrIAA27-RACE5'	5'-ATGCCATTAACCCAATGGCATACC-3'
TrIAA27-gene primer- F	5'-ATGTCTGTGCCAGTGGAAC-3'
TrIAA27-gene primer- R	5'-CTAGTTGCGACTTTTGCACT-3'
pCAMBIA1301-vector construct-F	5'-ATGGCGGAAACCTTTCTATT-3'
pCAMBIA1301-vector construct-R	5'-TTAAGCTTGAGGCTTCTCCCACTTG-3'
pSUPER1300- Vector construct-F	5'-CCAAATCGACTCTAGAATGTCTGTGCCAGTGGAACAAG-3'
pSUPER1300- Vector construct-R	5'-CCCTTGCTCACCATGGTACCGTTGCGACTTTTGCACTTTTCC-3'
TrIAA27 qRT-PCR-F	5'- CTCCTTCCATACTGGTCTCCTCCGC-3'
TrIAA27 qRT-PCR-R	5'- GCCCAAACATTAGGTGGTCTT-3'
Trβ-Actin qRT-PCR-F	5'-TTACAATGAATTGCGTGTTG-3'
Trβ-Actin qRT-PCR-R	5'-AGAGGACAGCCTGAATGG-3'

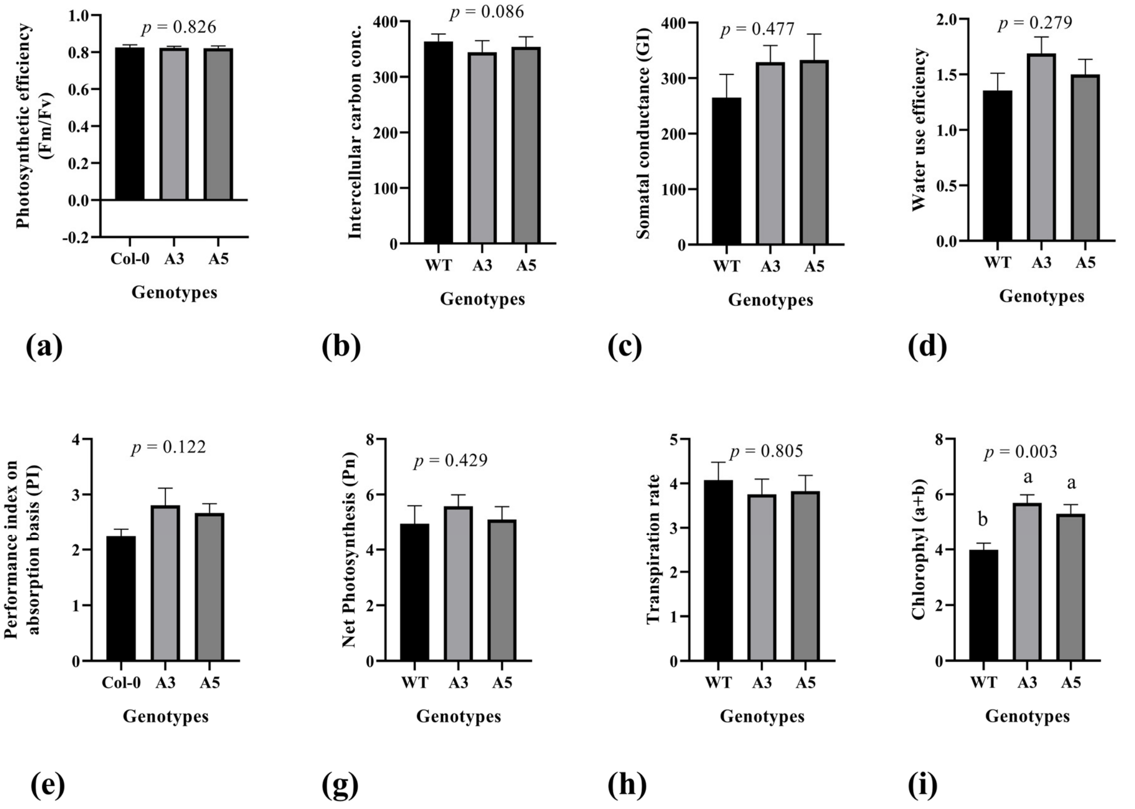

a

Supplementary Figure S1. Statistical comparison of photosynthesis-related traits of wild-type (WT) and *TrIAA27* overexpression (OE) lines of *Arabidopsis thaliana*. Different alphabet letters over bars show statistically significant differences (ANOVA, $p < 0.05$) among genotypes, and the error bar show standard error (SE). WT, OE3, and OE5 represent wild types, overexpression line 3 and overexpression line 5, respectively.
